# Lysophosphatidic Acid Pretreatment Attenuates Myocardial Ischemia/Reperfusion Injury in the Immature Hearts of Rats

**DOI:** 10.3389/fphys.2017.00153

**Published:** 2017-03-21

**Authors:** Haibo Chen, Si Liu, Xuewen Liu, Jinjing Yang, Fang Wang, Xiangfeng Cong, Xi Chen

**Affiliations:** State Key Laboratory of Cardiovascular Disease, Fuwai Hospital, National Center for Cardiovascular Diseases, Chinese Academy of Medical Sciences - Peking Union Medical CollegeBeijing, China

**Keywords:** lysophosphatidic acid, ischemia/reperfusion injury, apoptosis, glucose uptake

## Abstract

The cardioprotection of the immature heart during cardiac surgery remains controversial due to the differences between the adult heart and the newborn heart. Lysophosphatidic acid (LPA) is a small bioactive molecule with diverse functions including cell proliferation and survival via its receptor: LPA_1_–LPA_6_. We previously reported that the expressions of LPA_1_ and LPA_3_ in rat hearts were much higher in immature hearts and then declined rapidly with age. In this study, we aimed to investigate whether LPA signaling plays a potential protective role in immature hearts which had experienced ischemia/reperfusion (I/R) injury. The results showed that in Langendorff-perfused immature rat hearts (2 weeks), compared to I/R group, LPA pretreatment significantly enhanced the cardiac function, attenuated myocardial infarct size and CK-MB release, decreased myocardial apoptosis and increased the expression of pro-survival signaling molecules. All these effects could be abolished by Ki16425, an antagonist to LPA_1_ and LPA_3_. Similarly, LPA pretreatment protected H9C2 from hypoxia-reoxygenation (H/R) induced apoptosis and necrosis *in vitro*. The mechanisms underlying the anti-apoptosis effects were related to activation of the phosphatidylinositol 3-kinase (PI3K)/protein kinas B (AKT) signaling pathways as well as phosphorylation of the downstream effector of AKT, glycogen synthase kinase 3 beta (GSK3β), through LPA_1_ and/or LPA_3_. What's more, we found that LPA preconditioning increased glucose uptake of H9C2 subjected to H/R by the activation of AMP-Activated Protein Kinase (AMPK) but not the translocation of GLUT4. In conclusion, our study indicates that LPA is a potent survival factor for immature hearts against I/R injuries and has the potential therapeutic function as a cardioplegia additive for infantile cardiac surgery.

## Introduction

Similar cardioprotection methods have been used in adult and pediatric groups for many years (Turkoz, 2013). However, the immature heart is structurally, functionally, and metabolically different to the adult heart. For example, the immature heart mainly prefers to utilize glucose as its energy source rather than free fatty acids—which is the main substrate in the normal adult heart (~70%) (Onay-Besikci, 2006; Calmettes et al., 2015). Although the immature heart is more tolerant to ischemia, it is more vulnerable to reactive oxygen species after reperfusion than the adult heart (Starnes et al., 1997). Postoperative low cardiac output is still the major contributor to morbidity and mortality in pediatric cardiac operations (Imura et al., 2001). Nowadays the optimal myocardial protection strategy for newborns undergoing congenital heart surgery remains controversial. Therefore, it is necessary to explore an optimal myocardial protection strategy for the immature heart.

Lysophosphatidic acid (LPA, 1-acyl-2-lyso-sn-glycero-3-phosphate) is a simple endogenous bioactive phospholipid possessing diverse functions, such as: neurogenesis, angiogenesis, promoting cell migration and survival, as well as carcinogenesis (Mutoh et al., 2012). Acting as an extra-cellular signaling molecule, LPA exerts its function primarily through binding to and activating six known G protein–coupled receptors (GPCR_*S*_):LPA_1_–LPA_6_ (Hla et al., 2001; Choi et al., 2010). Our previous study indicated that the expression of LPA receptor 1 and 3 peaked on postnatal 7d and 4d, respectively, and decreased rapidly thereafter (Wang et al., 2012). Therefore, it reminds us of its potential role in myocardial protection during the neonatal period.

Apoptosis and necrosis are the primary pathologic factors which lead to cardiomyocyte damage during I/R (Ma et al., 2015; Wang et al., 2015). As a pro-survival factor, it is reported that LPA could inhibit the apoptosis of diverse cells, such as primary chronic lymphocytic leukemia cells, Schwann cells and renal tubular cells (Levine et al., 1997; Weiner and Chun, 1999; Deng et al., 2003). The mechanism involved in this anti-apoptosis procedure included activating the Hippo-pathway and the PI3K/AKT pathway, as well as inhibiting the mitochondrial intrinsic pathway; etc. (Hwang et al., 2014). Our earlier study also demonstrated the anti-apoptosis function in mesenchymal stem cells which had been subjected to ischemia (Chen et al., 2008). Thus, we were interested in ascertaining whether LPA has an anti-apoptosis role in immature heart I/R injury.

The enhanced glucose uptake and glycolysis have been shown to be cardioprotective (Zhang et al., 2015) under pathological conditions such as I/R. It is reported that LPA could stimulate the glucose uptake of myotube, adipocyte (Yea et al., 2008), cumulus-oocyte complexes (Thomson et al., 1996) and ovarian cancer cells (Kuwata et al., 2013). However, less is known about its role and functional significance in immature myocardial glucose uptake during I/R injury. Thus, we investigated whether LPA could improve the glucose uptake in cardiomyocytes subjected to IR injury.

Here, we hypothesized that LPA pretreatment may exert a cardioprotective effect on the immature heart after ischemia reperfusion injury by stimulating pro-survival signal pathways as well as the uptake of glucose. We aimed to investigate a potential cardioprotective agent for neonatal patients during cardiac surgery.

## Materials and methods

### Animals

Males, 2 weeks Sprague–Dawley rats, 25–30 g body weight (BW), were used for this study. Animals were fed a standard rat chaw and allowed to drink water *ad libitum*. They were housed in temperature-controlled (22–25°C) cages with a 12-h dark and 12-h light cycle. This study protocol was approved by the Fuwai Hospital Animal Care and Use Committee. The investigation conforms with the “Guide for the Care and Use of Laboratory Animals” published by the US National Institutes of Health (NIH Publication No. 85-23, revised 1996) and the “Regulation to the Care and Use of Experimental Animals” of the Beijing Council on Animal Care (1996).

### Langendorff model and study design

After being heparinized (3 IU/g BW) and anesthetized with 50 mg/kg BW of sodium pentobarbital by intraperitoneal injection, rats were subjected with a median sternotomy and the hearts were rapidly excised and placed in ice-cold heparinized K-H solutions. The time taken from opening of the chest to excision of the heart was 1–2 min. Immediately thereafter, the aorta was connected to a standard Langendorff apparatus and perfused with Krebbs–Henseleit (K-H) buffer solution (pH 7.4: in mM: NaCl 118; KCl 4.7; CaCl_2_ 2.0; MgSO_4_ 1.2; NaHCO_3_ 25; KH_2_PO_4_ 1.2; glucose 11.1), and continuously gassed with 95% O2 and 5% CO2 at pressure of 80 mmHg at 37°C. A pulmonary arteriotomy was performed to allow free drainage of coronary effluent. A water-filled latex balloon connected to a pressure transducer was inserted into the left ventricle to assess the LV function. Parameters, including heart rate (HR), left ventricle systolic pressure (LVSP) and left ventricular end-diastolic pressure (LVEDP), were continuously measured. The volume of the latex balloon was adjusted to set LVEDP to 0–10 mmHg during the stabilization phase.

**Figure 1:**
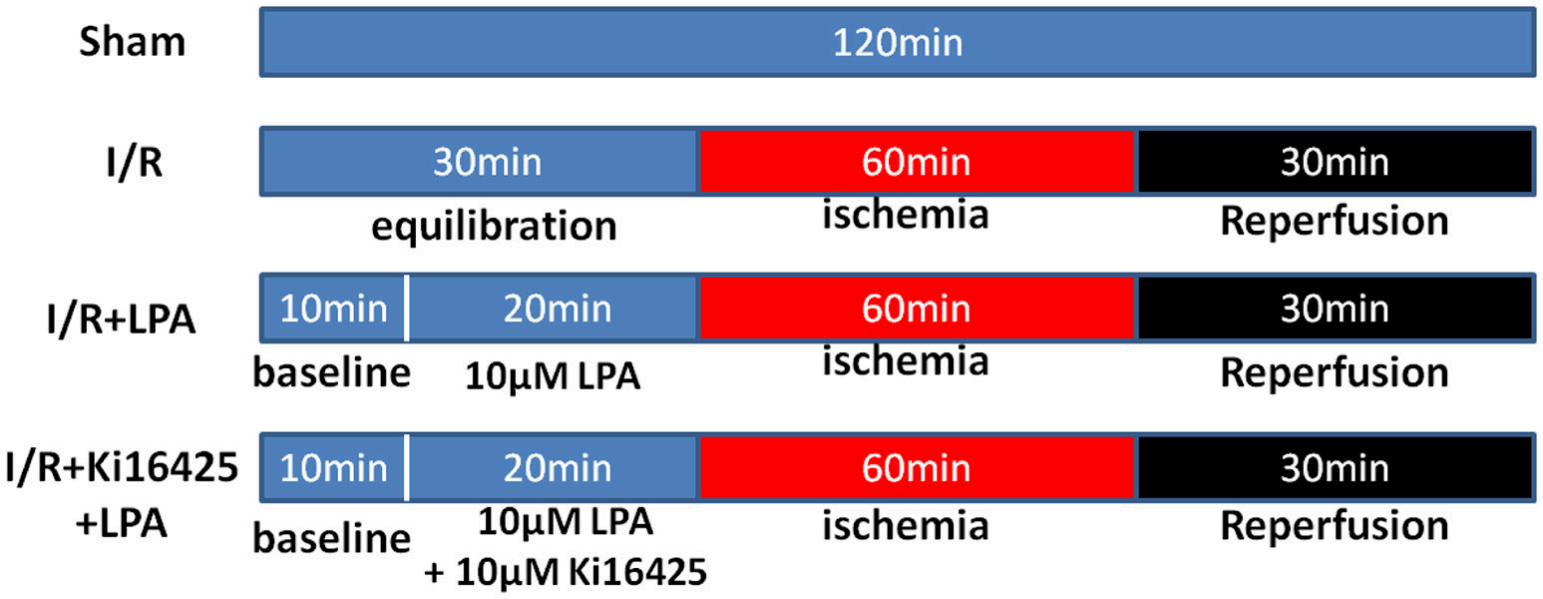
**A schematic representation of the experimental protocol**.

Rats were randomly assigned to one of four experimental groups. There were 6 rats in each group: (1) Sham group; (2) I/R group; (3) I/R+LPA group; (4) I/R+Ki16425+LPA group. The design of the experimental protocol is illustrated in Figure 1. All hearts were equilibrated for 10 min followed by a 20-min treatment period and subjected to 60 min of global ischemia by stopping the K-H buffer perfusion (maintaining 37°C), followed by 30 min of reperfusion. LPA (10 μM) was added into the perfusion fluid before ischemia and throughout reperfusion with or without ki16425 (10 μM).

### Assessment of infarct size

After 30 min of reperfusion, the isolated hearts were removed and cut into uniform sections of 2–3 mm thickness and incubated for 20 min in 0.1 M sodium phosphate buffer containing 1% 2,3,5-triphenyl tetrazolium chloride (TTC) (Sigma–Aldrich, St. Louis, MO, USA) at 37°C. Viable myocardium showed up as red, and the infarcted area showed up as pale white. The infarct size was calculated using Image-Pro Plus software, and was expressed as a percentage of the total heart size. The level of creatine kinase-MB (CK-MB) was measured from coronary effluent which was collected while reperfusion took place, in order to assess the infarct degree. The method of measurement was by spectrophotometry, using commercial assay kits (Roche, Indianapolis, IN, USA).

### TUNEL assay and assessment of caspases activity

The heart was fixed in neutral 10% formalin for 24 h, embedded in paraffin, and cut into 8 μm-thick slices. After de-paraffinating and re-hydratation, sections underwent antigen retrieval by boiling in sodium citrate solution for 2 min. TdT-mediated dUTP nick end labeling (TUNEL) staining was performed using a fluorescence detection kit (Roche, Indianapolis, IN, USA). Thereafter sections were covered with Mounting Medium with DAPI (Invitrogen, California, USA). Images were acquired using an Olympus fluorescence microscope and counted using the Image- Pro Plus software from 10 random fields at 400 × magnification. In order to examine caspase-3 activity, commercial kit reagents (Biovision Research Products, Mountain View, CA, USA), were used in accordance with the procedures outlined by the manufacturer. Samples of whole left ventricular homogenate were prepared and tissues were homogenized in the cell lysis buffer followed by centrifugation. The supernatant was collected and used for the assay. Protein concentration in the lysate was measured and 200 μg lysate protein was incubated in a 96-well plate with 2 × Reaction Buffer (50 μl) at 37°C in the dark. Caspase activity was monitored using a Microplate Reader at 405 nm.

### Cell culture

Embryonic rat heart derived H9C2 cells, from the cell bank at the Shanghai Institute for Biological Sciences, were cultured in L-DMEM (GIBCO, Grand Island, NY) supplemented with 10% FBS (fetal bovine serum, GIBCO) and antibiotics (50 U/mL penicillin and 50 μg/mL streptomycin, GIBCO) at 37°C in a 95% room air/5% CO2 incubator and sub-cultured when 80% confluence was reached. Before different treatments to H9C2, the medium would be replaced by serum-free DMEM when the H9C2 was grown to 80% confluence. To establish an I/R model *in vitro*, a Hypoxia-reoxygenation cell model was used in our study. Briefly, after serum-starvation for 18 h and then treating with different concentration of agents, H9C2 was subjected to hypoxia in a controlled hypoxic plastic chamber for 12 h. Reoxygenation was conducted in a normoxic incubator at 37°C after hypoxia for 4 h. For each evaluation, all experiments were conducted in triplicate.

### Evaluation of cell viability and apoptosis

Cell viability was measured using a quantitative colorimetric assay with thiazolyl blue tetrazolium bromide (MTT, AMResco), showing the mitochondrial activity of living cells. H9C2 cells (~3 × 10^4^) were seeded in 24-well plates. After drug treatment as indicated, cells were incubated with 300 μL MTT (final concentration 0.5 mg/mL) for 4 h at 37°C. The reaction was terminated through the addition of 200 μL DMSO. The MTT reaction products were determined by measuring the absorbance at 630 nm in a CHAMELEON micro-plate reader (HIDEX).

Hoechst 33258 (Invitrogen, California, USA) was used to detect the apoptosis of H9C2 in different groups. Briefly, H9C2 was stained with Hoechst 33258 dye solution (5 μg/mL) for 20 min at room temperature, in darkness, and then examined and photographed using a fluorescence microscope. Apoptotic cells were identified as previously described. The percentage of apoptotic cells was calculated as the ratio of the positive cell number to the total cell number by Image J.

### Flow cytometry

The amounts of apoptotic cells induced by hypoxia-reoxygenation were determined using an Annexin V-FITC apoptosis detection kit (SIGMA, USA), according to the manufacturer's instructions, as previously described. Cells in the initial stage of apoptosis were defined as annexin V (+)/propidium iodide (PI) (−), while late apoptotic cells were defined as annexin V (+)/PI (+). Briefly, H9C2 seeded in 6-wells plates were washed twice with cold PBS, then harvested and stained with 5 μl annexin V and 10 μl propidium iodide (PI) per 500 μl cells for 15 min at RT in the dark. Flow cytometric analyses were performed on a FACS Calibur Analyzer (BD). The apoptotic cells, including annexin V + /PI, were counted.

### 2-NBDG uptake of H9C2

H9C2 was incubated with 2-NBDG, which is a fluorescent glucose analog 2-(N-(7-nitrobenz-2-oxa-1, 3-diazol-4-yl) amino)-2-deoxyglucose, to investigate the glucose uptake ability. H9C2 was seeded in six-well plates. After hypoxia-reoxygenation—as in a previous description—cell culture was replaced by Kreb's buffer without glucose (NaCl [145 mM], KCl [5 mM], CaCl_2_ [6 mM], MgCl_2_ [1 mM], HEPES Na [25 mM], and NaHCO_3_ [10 mM], pH 7.4) and incubated with different concentrations of LPA and insulin for 20 min at 37°C. Thereafter, 2-NBDG (100 μM) (Invitrogen, California, USA) was supplemented at the same culture temperature for an additional 10 min in the dark. Fluorescence was measured in a FACSCalibur Analyzer (BD) by 488 nm (excitation wavelength) channel. Data was collected from 20,000 events for each measured sample.

### Western immunoblotting

After immunoblotting, the intensity of the immunoblot bands was detected utilizing a ChemiDoc XRS+ (Bio-Rad) instrument. Antibodies against the following proteins were purchased from Cell Signaling Technology: anti-Bax (1:1,000), anti-Bcl-2 (1:1,000), anti-GSK3beta (1:1,000), anti-phosphorylated GSK3beta (1:1,000), anti-AKT (1:1,000), anti-p-AKT (Ser473; 1:1,000), anti-pThr172-AMPKα (1:1,000), anti-GLUT4 (1:500) and anti-GAPDH (1:10,000; loading control) antibodies. In order to measure the amount of GLUT4 in the plasma membrane, the plasma membrane protein was separated from the cell by using a compartmental protein extraction kit (Millipore, USA).

### Statistical analysis

SPSS16.0 for Windows (SPSS Inc, Chicago, III) was used for data analysis. All values were expressed as mean ± standard error of the mean (SEM). For comparisons between groups, the one-way ANOVA or unpaired Student *T*-test, was used where appropriate. *Post hoc* testing used a Bonferroni Correction for multiple comparisons. If the test for normality failed or if the sample was <5, a Fisher Exact Test was used. A *P* < 0.05 was considered statistically significant.

## Result

### LPA pretreatment improved the cardiac function recovery of immature rats through LPA receptor 1/3 during I/R injury

**Figure 2:**
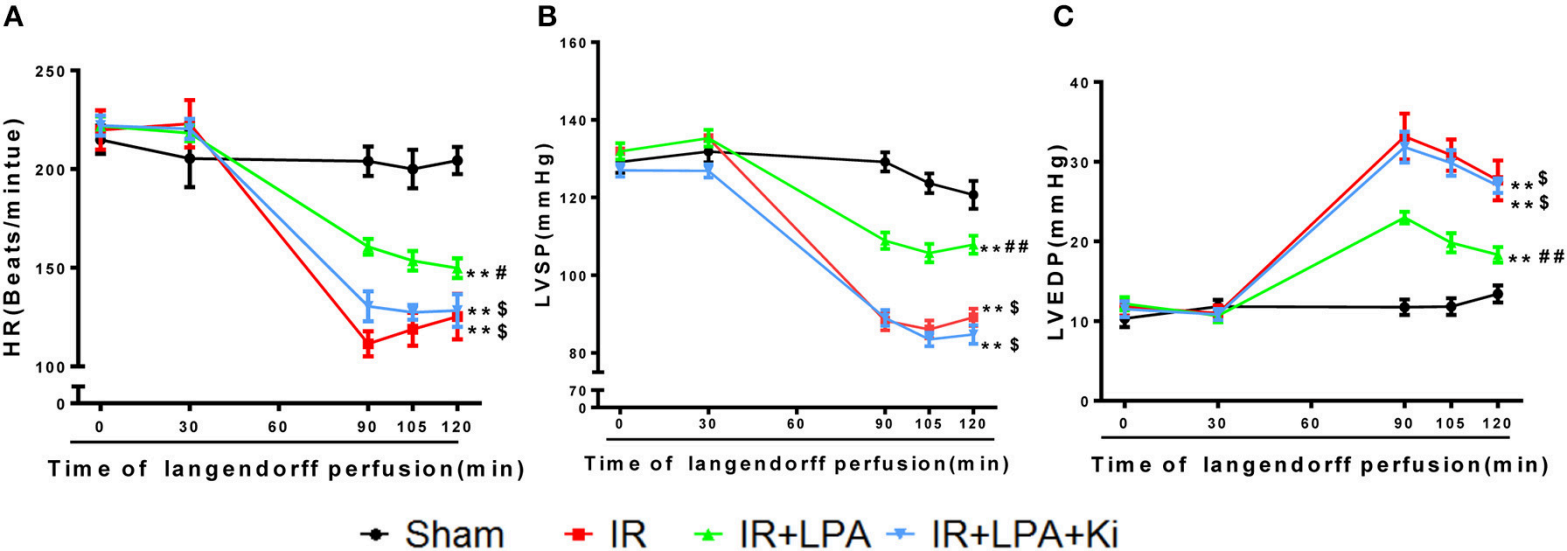
**LPA pretreatment improved the cardiac function recovery of immature rats through LPA receptor 1/3 during myocardial I/R injury. (A–C)** Hemodynamic parameters (HR, LVSP, LVEDP) before ischemia (0 and 30 min) and 90, 105, 120 min during reperfusion in each group. LVSP indicates left ventricular systolic pressure; LVEDP, left ventricular end diastolic pressure; HR, heart rate; IR, ischemia reperfusion. All values are expressed as mean ± SEM (*n* = 6 in each group). ^**^P < 0.01 vs. sham group; ^*##*^*P* < 0.01 vs. IR group; ^*$*^*P* < 0.05 vs. IR + LPA group.

There were no significant differences among the groups under basal conditions in cardiac LV function before the start of ischemia (Table 1). After 60 min of global ischemia, the I/R group exhibited a significant reduction in heart rate (HR) (Figure 2A) and left ventricle systolic pressure (LVSP) (Figure 2B) and a remarkable increase in LVEDP (Figure 2C) (*P* < 0.01 vs. sham group, respectively). Compared with the IR group, administration of LPA significantly enhanced the recovery of HR, LVSP and LVEDP within 30 min of reperfusion (Figures 2A–C). Furthermore, the administration of Ki16425 partially blocked the LPA-induced improvement in ventricular systolic and diastolic function after I/R (Figures 2A–C).

**Table 1:** **Baseline functional parameters in the Langendorff-perfused rat groups**.

	**SHAM (*n* = 6)**	**IR (*n* = 6)**	**LPA + IR (*n* = 6)**	**KI + LPA + IR (*n* = 6)**
BW (g)	25.4 ± 0.4	26.1 ± 0.3	25.9 ± 0.5	27.4 ± 0.2
HW (mg)	130.7 ± 6.1	125.6 ± 5.4	127.9 ± 6.3	125.4 ± 4.2
LVSP (mmHg)	92 ± 5.7	93 ± 6.4	92 ± 5.3	90 ± 6.6
LVEDP (mmHg)	7.9 ± 0.7	7.8 ± 1.1	7.5 ± 0.6	7.7 ± 0.4
H R (beats/min)	315 ± 14	322 ± 16	319 ± 15	317 ± 13
CFR (ml/min)	2.1 ± 0.2	2.2 ± 0.5	2.0 ± 0.2	2.3 ± 0.4

*All the values are expressed as mean ± SEMs; BW, body weight; HW, heart weight; LVSP, left ventricular systolic pressure; LVEDP, left ventricular diastolic pressure; HR, heart rate; CFR, coronary flow rate*.

### LPA pretreatment attenuates myocardium infarct size and apoptosis through LPA receptor 1/3 after I/R injury

**Figure 3:**
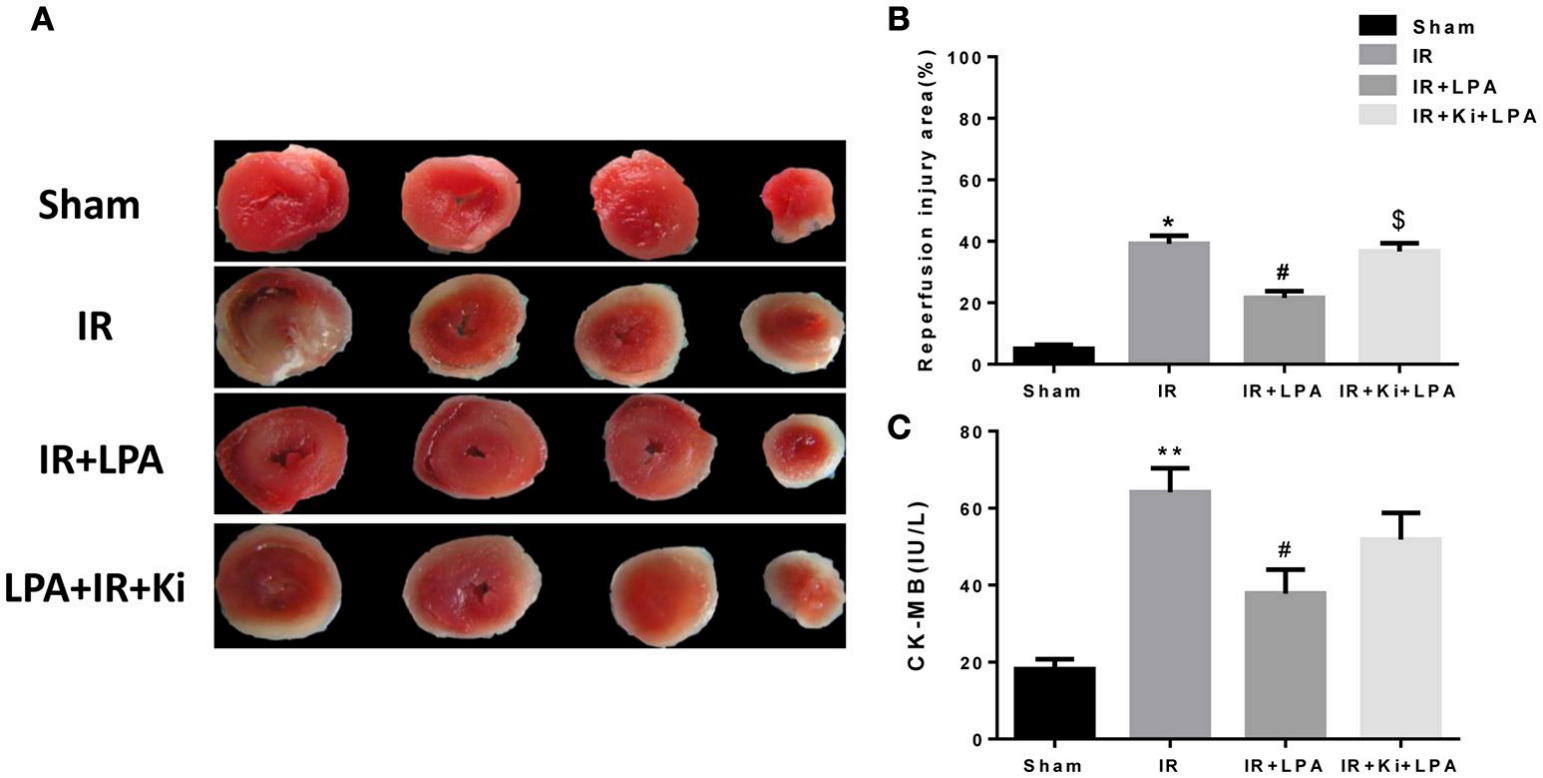
**LPA pretreatment attenuates myocardium infract size and CK-MB release through LPA receptor 1/3 after I/R injury. (A)** Representative images of TTC staining in each group (*n* = 6, 4 slices per heart). The red-stained areas indicate viable tissue, and the non-stained pale areas indicate infarct tissue. **(B)** Infarct size in each group. **(C)** Creatinine kinase, MB isoenzyme (CK-MB) activity in each group. All values are expressed as mean ± SEM (*n* = 6 in each group). ^*^P < 0.05 vs. sham group; ^**^P < 0.01 vs. sham group, ^#^*p* < 0.05 vs. IR group; ^*$*^*P* < 0.05 vs. IR + LPA (10 μmol/L) group.

The infarct size determined by TTC staining was significantly lower in the IR+LPA group than in the IR group (Figures 3A,B). However, the infarct-limiting effect of LPA was abolished through pretreatment with Ki16425 (Figures 3A,B). The released CK-MB was also measured in each experimental group to determine the degree of myocardial injury. Global ischemia followed by reperfusion significantly increased CK-MB levels, and LPA pretreatment significantly decreased the release of CK-MB compared with IR group (Figure 3C). Similarly, these effects were also partially abolished by Ki16425 (Figure 3C).

**Figure 4:**
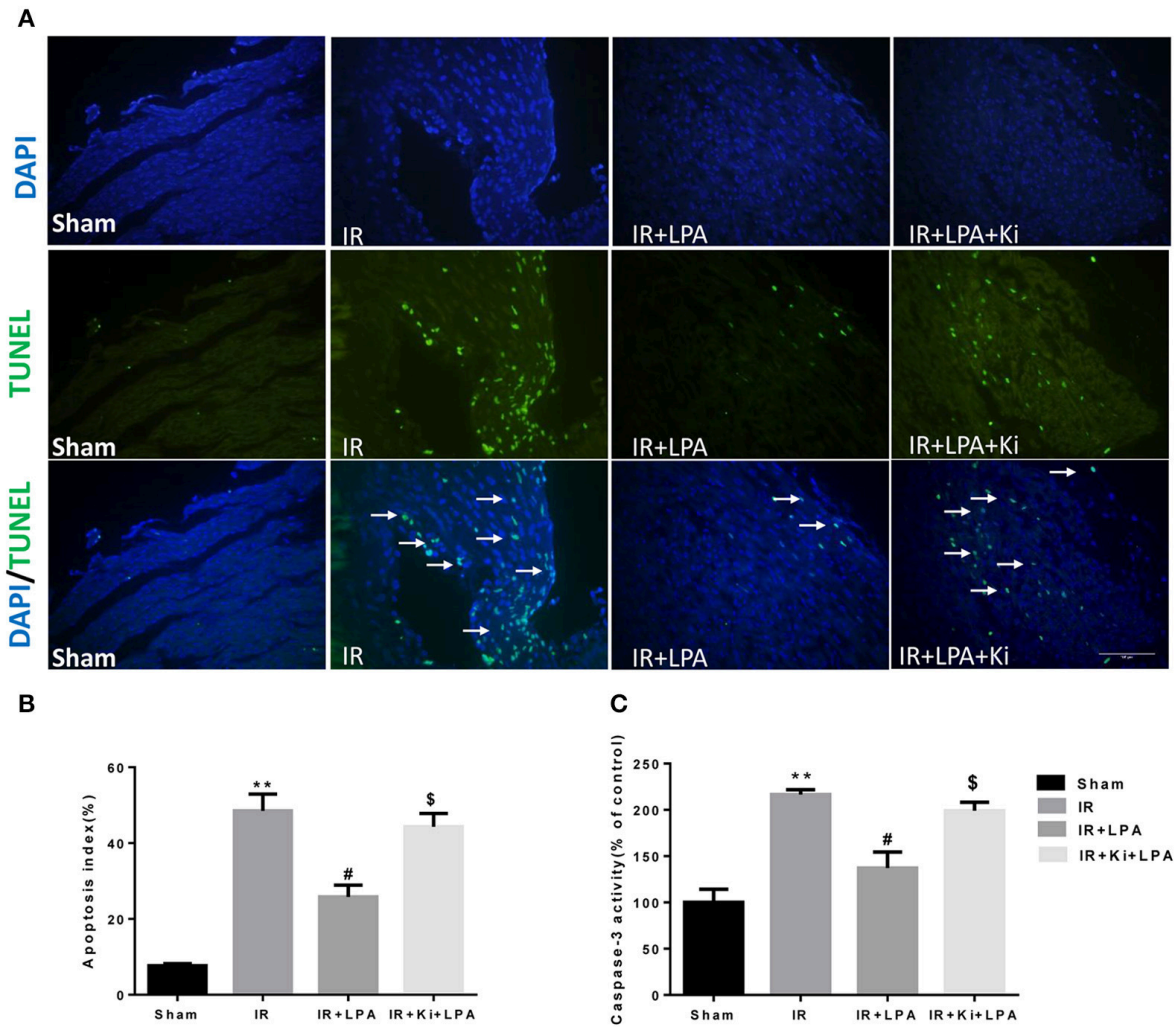
**LPA pretreatment attenuates the apoptosis of myocardium caused by ischemia and reperfusion through LPA receptor 1/3. (A)** Representative photographs of myocardial apoptosis by TUNEL assay in each group. The TUNEL positive cells are indicated by arrows. **(B)** Quantitative data on the percentage of TUNEL-positive cells to total number of cells in each group. **(C)** Caspase-3 activity in each group. All values are expressed as mean ± SEM (*n* = 6 in each group). ^**^p < 0.01 vs. sham group, ^#^*P* < 0.05 vs. IR group; ^*$*^*P* < 0.05 vs. IR + LPA group.

As displayed in Figures 4A,B, IR significantly increased the percentage of TUNEL-positive cells. This increase was attenuated through pretreatment with LPA, and Ki16425 attenuated the anti-apoptotic effects of LPA. Consistent with the TUNEL data, IR significantly increased the activity of infantile myocardial caspase-3, which was rescued by pretreatment with LPA (Figure 4C). Compared with the LPA group, co-administration of LPA and Ki16425 significantly increased the apoptosis index and the activity of caspase-3 (Figures 4B,C), which indicated that LPA exerted its anti-apoptotic effects by coupling to its receptor 1/3.

### LPA pretreatment increased the expression of pro-survival signaling molecules after IR injury

**Figure 5:**
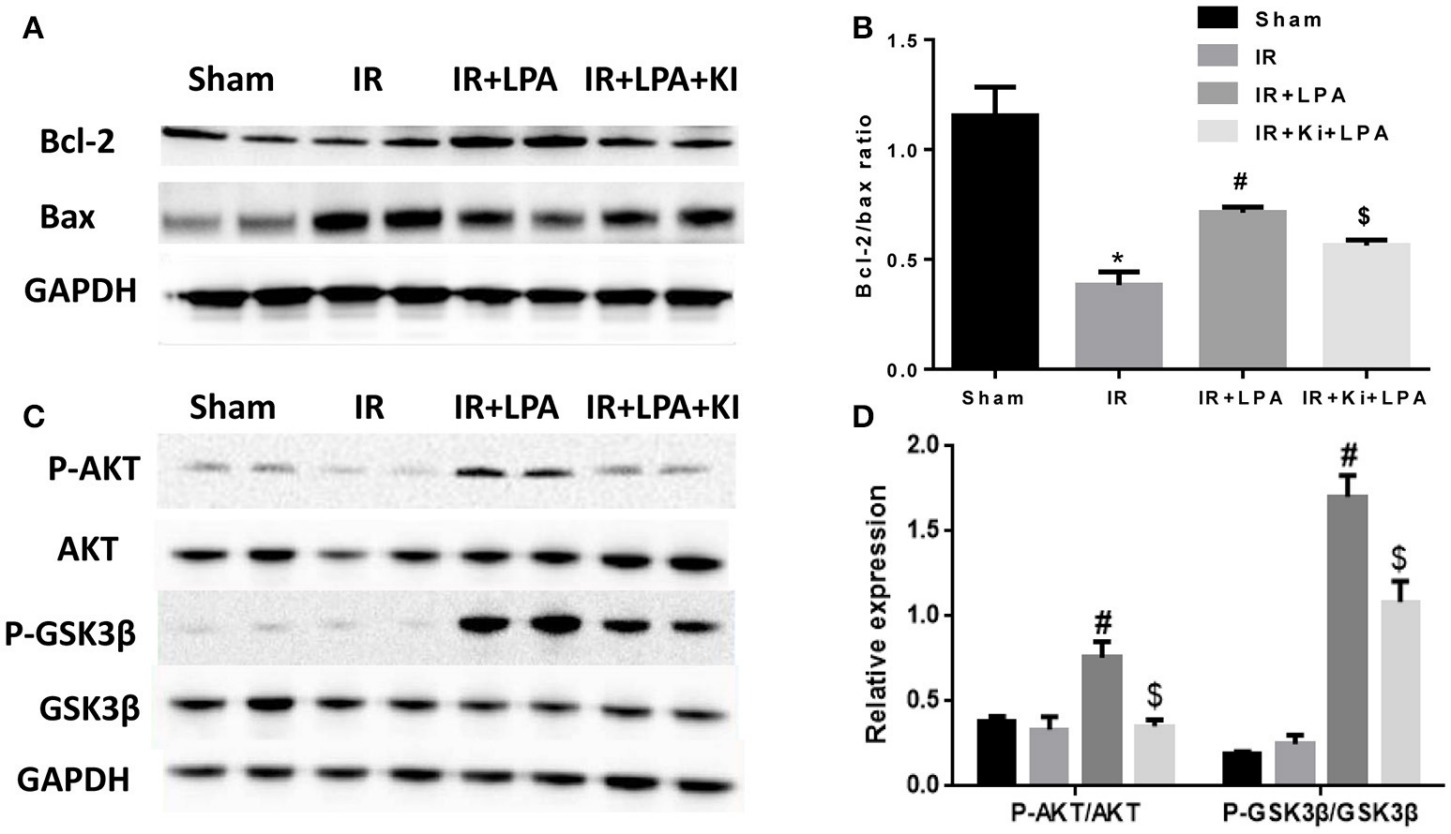
**LPA pretreatment increased the expression of pro-survival signaling molecules after IR injury. (A,B)** Western blots analysis of the protein level of Bcl-2 and Bax. **(C,D)** Western blots analysis of protein level of phosphorylated AKT, and phosphorylated GSK3b. All values are expressed as mean ± SEM (*n* = 3 in each group). IR, ischemia reperfusion; Ki, ki16425. ^*^P < 0.05 vs. sham group; ^#^*P* < 0.05 vs. IR group; ^*$*^*P* < 0.05 vs. IR + LPA group.

Furthermore, the expression of classic anti-apoptotic (Bcl-2) and pro-apoptotic proteins (Bax) were examined. IR significantly reduced Bcl-2/Bax relative expression, and pretreatment with LPA increased Bcl-2/Bax relative expression (Figures 5A,B). In addition, western blot analyses of heart tissue indicated that IR induced a statistically significant reduction in the phosphorylation of AKT. LPA pretreatment markedly elevated the phosphorylation of AKT and its downstream molecule GSK-3β. However, those effects were abolished by Ki16425 treatment (Figures 5C,D). These results indicate that the cardioprotective effect of LPA is attributable to the activation of the AKT signaling pathway through the LPA receptor 1/3.

### Pretreatment with LPA attenuates hypoxia-reoxygenation induced apoptosis and necrosis in cultured H9C2 *in vitro*

**Figure 6:**
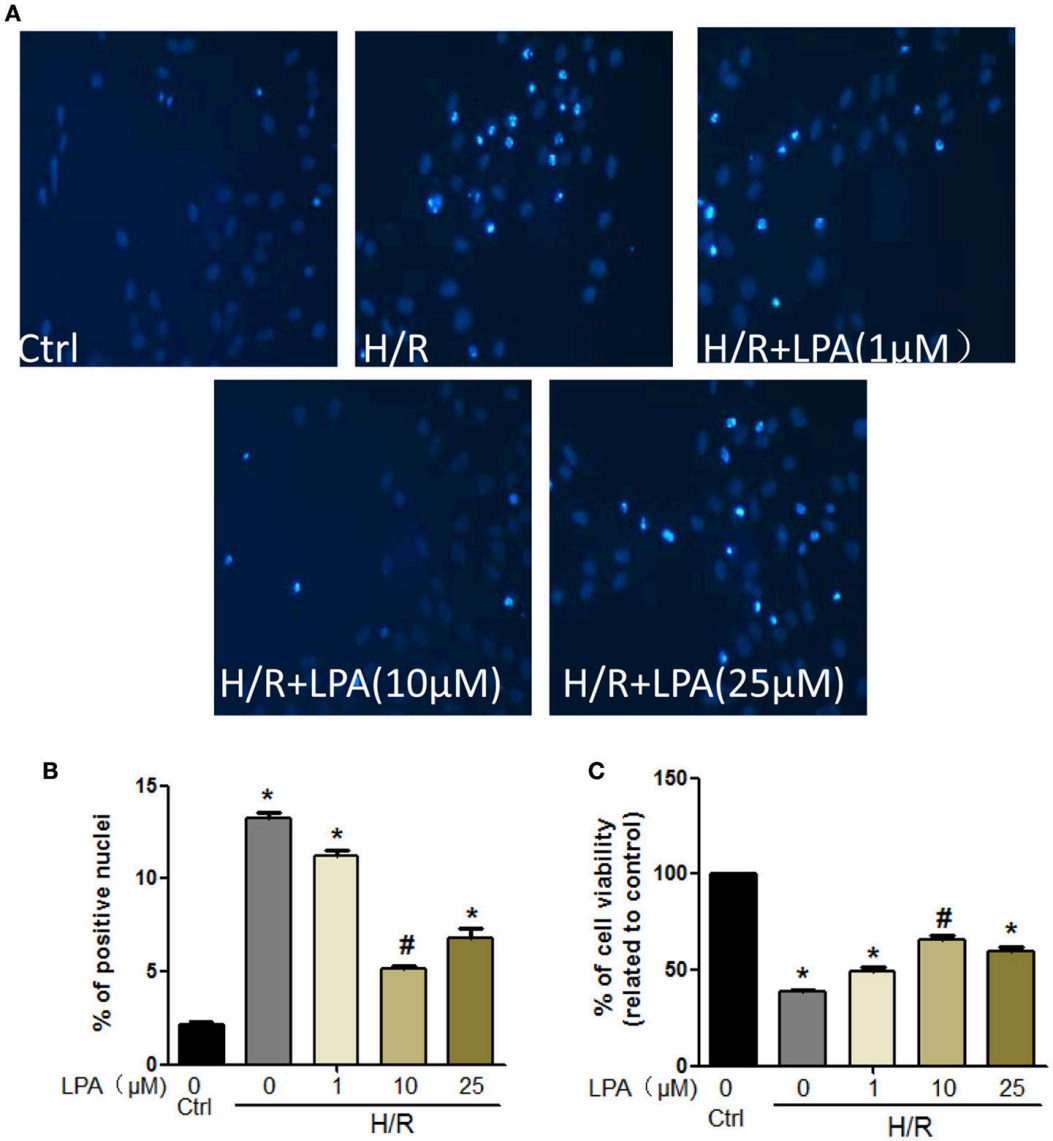
**LPA administration protects H9C2 from H/R induced cell apoptosis and death. (A,B)** Representative images and quantitative data on H9C2 apoptosis obtained by Hochest staining (*n* = 3 in each experiment). **(C)** Cardiomyocytes viability after pretreatment with LPA (1, 10, or 25 μmol/L) with or without suffering from H/R detected by MTT analysis. All values are expressed as mean ± SEM. ^*^P < 0.05 vs. control; ^#^*P* < 0.05 vs. H/R.

**Figure 7:**
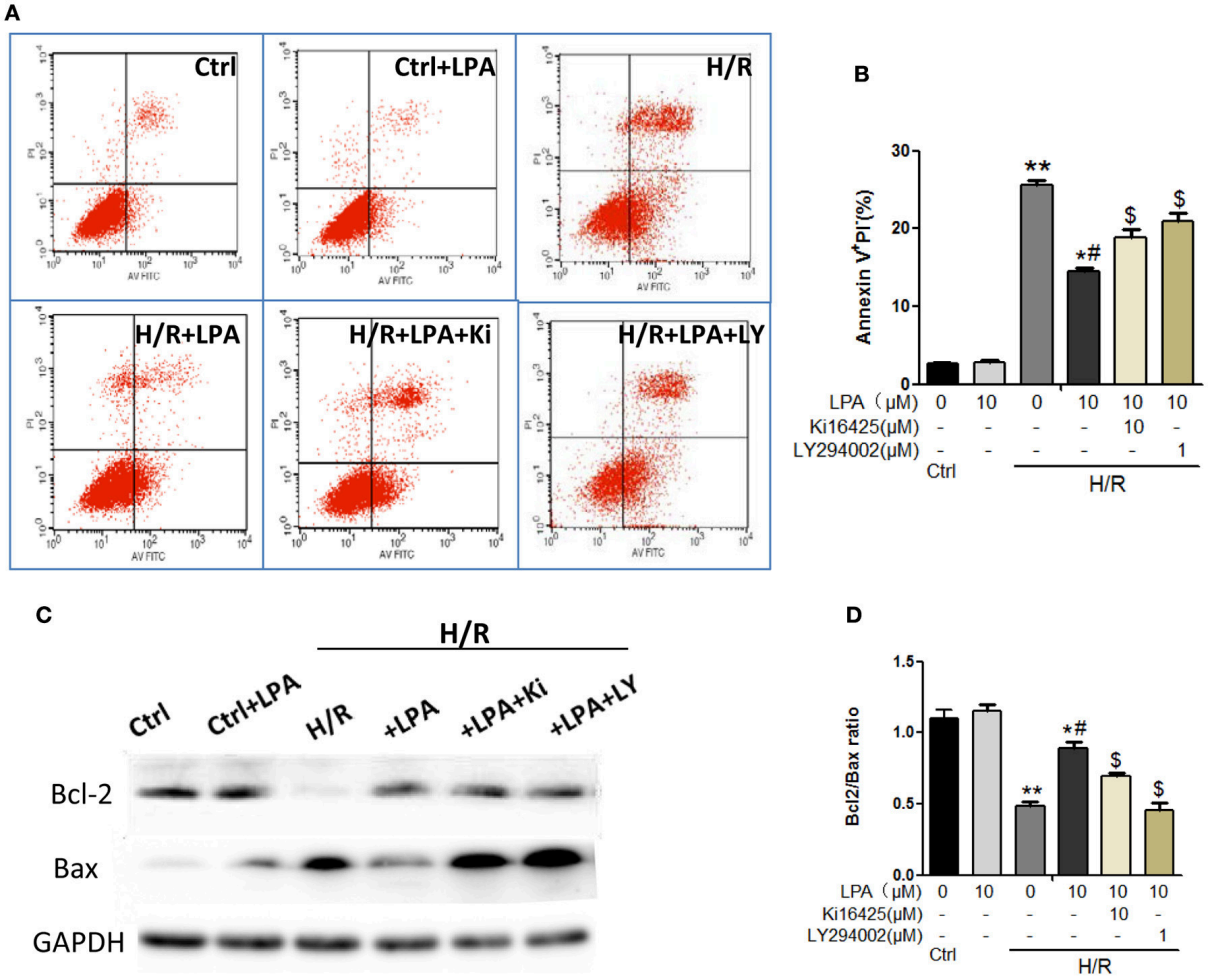
**LPA pretreatment attenuates H/R induced apoptosis and necrosis in cultured H9C2 ***in vitro***. (A)** Representative images of H9C2 cardiomyocytes apoptosis detected by flow cytometry after staining with Annexin V and propidium iodide (PI) in each group. **(B)** Quantifications of the apoptotic cardiomyocyte (Annexin V+/PI-)were presented as the percentage of total cells. **(C,D)** Western blots analysis of Bax and Bci-2. AII values are expressed as mean ± SEM (*n* = 3 in each group). ^*^P < 0.05 vs. ctrl group; ^**^P < 0.01 vs. Ctrl group; ^#^*P* < 0.05 vs. H/R group; ^*$*^*P* < 0.05 vs. H/R + LPA group.

Cell viability was decreased when H9C2 suffered from hypoxia-reoxygenation (H/R) which was a cell model of ischemia reperfusion (IR), as shown by the MTT assay. This change was rescued by LPA pretreatment which was administered in a dose-dependent manner (Figure 6C). Moreover, H/R increased the number of apoptotic H9C2, as shown by the Hoechst staining (Figure 6A). LPA preconditioning significantly reduced the extent of H9C2 apoptosis compared with the H/R group (Figure 6B). Apoptosis/necrosis was also evaluated using the Annexin V-FITC Apoptosis Detection Kit. The percentage of apoptosis and necrosis cells in the IR group was significantly higher than the control group (Figures 7A,B). LPA preconditioning significantly protected H9C2 from H/R induced apoptosis. However, when LPA administration was combined with PI3K/AKT inhibitor LY294002, the anti-apoptosis effect of LPA was negated. Consistently, the results of western blot indicated that IR significantly reduced Bcl-2/Bax relative expression, and pretreatment with LPA increased Bcl-2/Bax relative expression (Figures 7C,D).

### LPA preconditioning increased the glucose uptake in H9C2 cells subjected to hypoxia-reoxygenation

**Figure 8:**
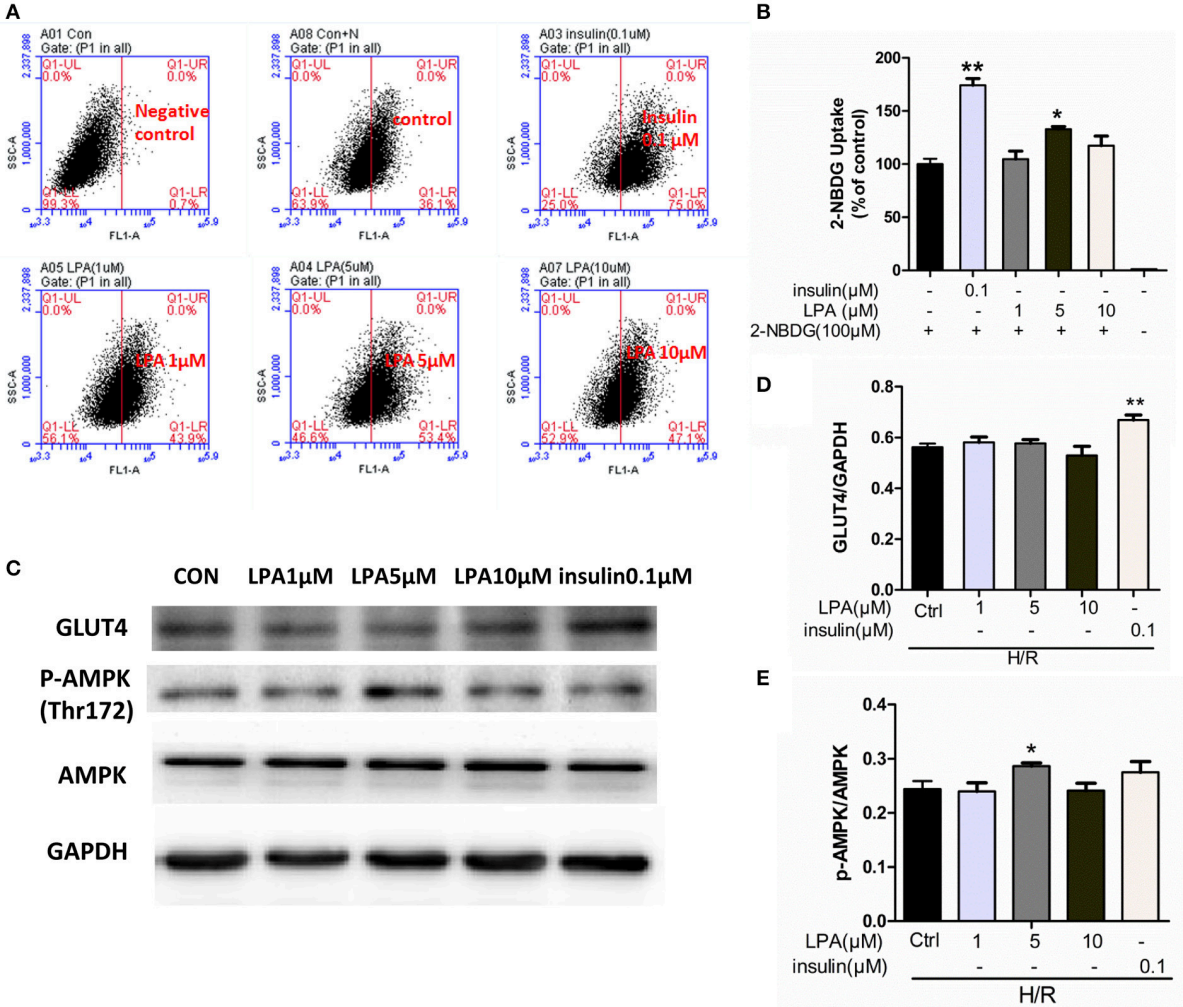
**LPA pretreatment increased glucose uptake of H9C2 suffered from H/R. (A)** Representative flow cytometry assay image of 2-NBDG uptake. **(B)** The ratio of 2-NBDG uptake in each group. **(C)** H9C2 was stimulated with different concentration LPA to determine the phosphorylation level of AMPK at Thr 172 and GLUT4 after H/R by performing Western blotting. **(D)** GLUT4 levels were normalized to GAPDH. **(E)** Phospho-AMPK levels were normalized to total AMPKa. All values are expressed as mean ± SEM. ^*^P < 0.05 vs. Ctrl; ^**^P < 0.01 vs. Ctrl (H/R+0 μM LPA) group.

To investigate the effects of LPA preconditioning on cardiomyocyte glucose uptake, the uptake of fluorescent glucose (2-NBDG) was studied. As shown in Figures 8A,B, after hypoxia 12 h, LPA (5 μM) significantly increased 2-NBDG uptake in H9C2. In the study, the Insulin (0.1 μM) treatment group was used as a positive control. As expected, insulin increased glucose uptake faster than LPA.

### The effect of LPA on GLUT4 translocation and AMPK pathway

As shown in Figures 8C,D, there was no significant difference between the control group and the LPA preconditioning group on GLUT4 translocation from the cytoplasm to the membrane. However, the insulin treatment group—which is a positive control—showed a significant increase of GLUT4 translocation. In addition, the role of AMP-Activated Protein Kinase (AMPK) was investigated in this procedure. As shown in Figures 8C,E preconditioning with 5 μM LPA significantly enhanced the phosphorylation of AMPK (*P* < 0.05), while insulin did not. These results suggested that LPA was involved in the activation of AMPK pathway but not the translocation of GLUT4.

## Discussion

LPA, a bioactive molecule released by activated platelets under pathological conditions, is involved in cardiovascular disease (Abdel-Latif et al., 2015). Our previous study has documented that LPA protects mesenchymal stem cells against ischemia-induced apoptosis (Chen et al., 2008) and LPA_1_ and LPA_3_ are highly expressed during the neonatal period (Wang et al., 2012). However, little is known about the role of LPA on immature hearts subjected to I/R injury. This study demonstrated for the first time that LPA pretreatment significantly improved cardiac function recovery in immature hearts following ischemia and alleviated H/R injury in cultured H9C2. In addition, the effects were dependent on the activation of LPA receptor 1 and/or 3. The underlying protection mechanism may be activating an anti-apoptotic pathway and improving the uptake of glucose. These results imply a protective effect of LPA in ischemia-reperfusion (I/R) injury of immature hearts.

It is reported that current cardioplegic protection techniques used in pediatric cardiac surgery do not take age into consideration (Starnes et al., 1997; Imura et al., 2001). Experimental studies have suggested that immature hearts are resistant to ischemia, and recover from hypoxia more efficiently than adult hearts (Liaw et al., 2013). However, compared to adults, infants experienced significantly more reperfusion injury and worse clinical outcomes (Starnes et al., 1997). In addition, immature hearts responded less to most pharmacological and physiological stimuli. Therefore, more specific cardiac cardioplegia is necessary and meaningful during pediatric cardiac surgery. This study demonstrated (using a Langendoff model) that LPA pretreatment could rescue the immature rat heart from left ventricular dysfunction caused by 1 h ischemia and 30 min reperfusion.

Wu et al. (2015) reported that cardiomyocyte apoptosis directly resulted in heart dysfunction during IR injury (Peng et al., 2011). Moreover, immature myocardium is more sensitive to oxidative stress (Umansky et al., 1995). In our study, it was found that I/R injury resulted in increasing apoptosis and myocardial infarction size, which is consistent with the results of other studies (Zhao, 2004; Ravingerová et al., 2013; Wang et al., 2015). LPA has been reported to inhibit apoptosis of various cells, such as cervical cancer cells, bone mesenchymal stem cells, human dental pulp cells, etc. (Pan et al., 2014; Sui et al., 2015). It was demonstrated here that LPA inhibits apoptosis induced by ischemia reperfusion injury in immature rat hearts and hypoxia-reoxygenation in H9C2. Furthermore, the signaling pathways involved in LPA-induced anti-apoptotic effects were investigated. PI3K/AKT activation has been reported to be involved in left ventricular functional reserve, reduction of apoptotic cardiomyocytes and limitation of infarct size (Fujio et al., 2000). This study revealed that LPA significantly increased the phosphorylation of AKT, as well as the ratio of Bcl-2/Bax. Furthermore, in the I/R cell model, the AKT inhibitor eliminated the anti-apoptotic effects of LPA. In the present study, it was observed that LPA caused a significant increase in the phosphorylation of GSK-3β at Ser9, which is an inactive form of GSK-3β. Hence, this predicted that inhibition of downstream GSK3β may have contributed to LPA-induced cardioprotection by enhancing the Bcl-2/Bax ratios (Juhaszova et al., 2009). De Sarno, et al, reported AKT activation improves cell survival through phosphorylation of GSK-3β, and the inhibition of GSK-3β reduces infarct size following focal cerebral ischemia *in vivo* (De Sarno et al., 2002). Similarly, our data suggested that PI3K/AKT-mediated deactivation of GSK-3β is, at the very least, partially responsible for the anti-apoptotic effects of LPA.

As subfamilies of G-protein-coupled receptors (GPCRs), LPA receptors mediate a wide range of cellular functions, including: inducing cell proliferation; regulating cell differentiation and survival; and suppressing cell apoptosis, etc. (Goetzl and An, 1998; Choi et al., 2010). Previous reports indicated that LPA protects intestinal epithelial cells from apoptosis through a mechanism involving LPA_2_/Erk/Bcl-2 signaling (Deng et al., 2002). However, in our animal model study, Ki16425 administration before LPA preconditioning abolished cardiac recovery in infantile hearts after I/R, and also eliminated the anti-apoptotic effects by blockage of AKT phosphorylation. The *in vitro* cell model also suggested that anti-apoptotic effects were blunted by Ki16425. Our data suggested that LPA protected immature hearts against IR injury through LPA_1_ and/or LPA_3_.

A shift in myocardial metabolism from fatty acid to glucose occurs when hearts suffer from myocardial I/R, which is more oxygen efficient and prevents deleterious effects (Lou et al., 2013; Xue et al., 2015). Therefore, improvement of glucose uptake will benefit the recovery of the infarcted heart. This study indicated that LPA stimulated glucose uptake when H9C2 was subjected to H/R. However, a significant change of GLUT4 content in cytomembrane was not found. In addition, we investigated the phosphorylation level of AMPK at Thr^172^ which plays a key role in regulating cellular energy metabolism. Indeed, we finally found that Phospho-AMPK levels increased after LPA treatment. Consistently, the phosphorylation of AMPK by LPA administration was also founded in LPA-induced cell migration in ovarian cancer cells (Kim et al., 2011) and the proliferation of diploid fibroblasts (Kwon et al., 2010). Many papers have previously stated that the activation of AMPK may result in stimulating GLUT4 translocation and enhancing glycolysis (Lee et al., 2015; Liang et al., 2015). Nevertheless, other studies indicated that AMPK activation did not redistribute GLUT4 to the sarcolemmal membrane (Lee et al., 2014). Taking this into consideration, we concluded that LPA preconditioning could increase the glucose uptake when H9C2 suffered from long-term H/R through activating AMPK but independent of GLUT4 translocation.

Here we should confess that there are some limitations in this study. The Langendorff heart perfusion system used in this study is a classic model to evaluate the effect of drugs on cardiac function *ex vivo*. However, Langendorff model cannot mimic the *in vivo* environment which contains mechanical and neurohumoral regulation. The *in vivo* model by left anterior descending (LAD) ligation and re-opening is the most representative of the clinical condition of myocardial ischemia/reperfusion (I/R) injury (Chang et al., 2012). As a result, further studies would be required to clarify the influence of LPA on myocardial infarction *in vivo*.

In conclusion, LPA preconditioning could prevent oxidative stress induced cardiomyocyte apoptosis and increase glucose uptake, finally improving the functional recovery of infantile hearts after ischemia reperfusion. It also showed that the effects were mediated through the LPA receptor 1 and/or 3/AKT/GSK3βpathways. These results clearly indicated that LPA could potentially provide an effective therapeutic benefit for infantile cardiac surgery as a cardioplegia additive.

## Author contributions

Designed the experiments: XChen, and XCong; Performed the experiments: HC, SL, XL, and JY; Analyzed the data: HC, SL, and FW; wrote the manuscript: HC, and SL; Revised the manuscript: XChen.

### Conflict of interest statement

The authors declare that the research was conducted in the absence of any commercial or financial relationships that could be construed as a potential conflict of interest.
